# Hemagglutination in gill capillaries of sheepshead, *Archosargus probatocephalus* (Perciformes: Sparidae), infected by a myxosporidean

**DOI:** 10.1590/S1984-29612022001

**Published:** 2022-01-14

**Authors:** Carlos Azevedo, Graça Casal, Emerson Carlos Soares, Elsa Oliveira, Sónia Rocha, Mike Hine, Themis Jesus Silva

**Affiliations:** 1 Laboratory of Cell Biology, Institute of Biomedical Sciences – ICBAS, Universidade do Porto – UP, Porto, Portugal; 2 Laboratory of Animal Pathology, Interdisciplinary Centre of Marine and Environmental Research – CIIMAR, University of Porto - UP, Matosinhos, Portugal; 3 Toxicology Research Unit – TOXRUN, University Institute of Health Sciences, Cooperativa de Ensino Superior Politécnico e Universitário – CESPU, Gandra, Portugal; 4 Laboratório de Aquicultura e Análise de Água – LAQUA, Universidade Federal de Alagoas – UFAL, Campus de Engenharias e Ciências Agrárias – CECA, Rio Largo, AL, Brasil; 5 Instituto de Investigação e Inovação em Saúde, i3S Universidade do Porto, Porto, Portugal; 6 Currently retired, Fouras, France

**Keywords:** Erythrocyte agglutination, *Henneguya* sp., ultrastructure, Aglutinação eritrocitária, *Henneguya* sp., ultraestrutura

## Abstract

During a survey Myxozoa, four specimens of the sheepshead (18 ± 1.5 cm and 59 ± 2.5 g) (*Archosargus probatocephalus*) were collected in the Ipioquinha river (Maceió/AL). Transmission electron microscopy observations revealed erythrocyte agglutinations in gill capillaries located near spherical cysts containing myxospores of the genus *Henneguya*. This hemagglutination partially or totally obstructed the gill capillaries. Erythrocytes occurred in close adherence to each other, with a closed intercellular space. A few lysed erythrocytes were observed among agglutinated cells. The reduced lumen of the capillaries was partially filled with amorphous dense homogenous material adhering to the erythrocytes. In addition, heterogeneous masses of irregular lower electron density were observed in the reduced channel of the capillary. The agglutinated erythrocytes appeared dense and homogenous, lacking cytoplasmic organelles. The nuclei had the appearance of normal condensed chromatin masses, generally without visible nucleoli. This occurrence of hemagglutination only in the capillaries located in close proximity to the developing myxozoan cysts suggests that parasite development may be a factor triggering erythrocyte agglutination. This is supported by previous experimental studies that showed a probable correlation between parasitic infections and hemagglutination. Nonetheless, further studies are necessary in order to better understand the physicochemical processes involved in this phenomenon.

## Resumo

Durante pesquisa de mixozoários foram coletados quatro espécimes do peixes sargo-de-dente (18 ± 1.5 cm e 59 ± 2.5 g) (*Archosargus probatocephalus*), no rio Ipioquinha (Maceió/AL). Observações por microscopia eletrônica de transmissão revelaram aglutinação de eritrócitos em capilares branquiais localizados próximos a cistos esféricos, contendo mixosporos do gênero *Henneguya*. Essa hemaglutinação obstruiu parcial ou totalmente os capilares branquiais. Os eritrócitos apareceram em forte aderência entre si, com espaço intercelular fechado. Foram observados poucos eritrócitos lisados entre as células aglutinadas. O lúmen reduzido dos capilares foi parcialmente preenchido com material homogêneo denso amorfo aderido aos eritrócitos, além de massas livres heterogêneas de densidade eletrônica baixa e irregular observadas no canal reduzido dos capilares. Os eritrócitos aglutinados pareciam densos e homogêneos, sem organelas citoplasmáticas. Os núcleos apareceram como massas normais de cromatina condensada, geralmente sem nucléolos visíveis. A ocorrência de hemaglutinação apenas nos capilares, localizados nas proximidades dos cistos mixozoários, sugere que o desenvolvimento parasitário pode ser um fator desencadeante da aglutinação eritrocitária. Isso é corroborado por estudos experimentais anteriores que mostraram uma provável correlação entre infecções parasitárias e hemaglutinação. No entanto, novos estudos são necessários para melhor compreender os processos físico-químicos envolvidos neste fenômeno.

## Introduction

Fish erythrocytes have been reported to be sensitive to environmental biotic and abiotic factors and their morphological evaluation has been used as a bioindicator for these factors (Ahmed et al., 2020; Galeotti et al., 2015; Witeska, 2013). Hematology is considered to be a powerful tool for understanding the health of aquatic organisms, in both wild and captive species (Maciel et al., 2016).

These environmental factors, as well as morphological alterations and agglutination of erythrocytes, have been experimentally demonstrated to have an influence on several animal species, including fish (Ahmed et al., 2020; Galeotti et al., 2015; Gupta & Poddar, 2014; Larsen & Mellergaard, 1984; Witeska, 2013). Nonetheless, little is known about the natural occurrence of erythrocyte agglutination (EAg) in the gill capillaries of fish species.

Experimental studies on EAg have shown that its occurrence depends on several factors, including antigens, antibodies, electrical properties of red blood cells, parasitism and environmental factors (Ahmed et al., 2020; Gupta & Poddar, 2014; Larsen & Mellergaard, 1984; Witeska, 2013). For instance, how toxicity of sodium fluoride has been reported to alter the morphology of erythrocytes and also the activity of antioxidant enzymes (Gupta & Poddar, 2014). Experimental studies have shown that trout (*Salmo gairdnerii = Salmo mykiss*) erythrocytes may agglutinate as a response to infection by strains of marine *Vibrio* species (Larsen & Mellergaard, 1984; Trust et al., 1981). Hemagglutination and hemolytic activity has also been demonstrated in erythrocytes of *Carassius auratus* in the presence of the pathogen *Aphanomyces piscicida* (Kurata et al., 2000). Several other experimental studies have correlated the activity of pathogenic agents with the occurrence of hemolytic, hemagglutinating and destructive activity in fish (Tamm, 1952; Yanuhar et al., 2019). Pathogen activity can affect blood cells and be a catalyst for the development of anemia in infected fish (Kurata et al., 2000). Stress is also a factor contributing to a significant increase in hemagglutination in specimens of *Sparus aurata* (Salati et al., 2016). Such stress can be triggered by several factors, including infection with microparasites.

The aim of the present study was to provide the first description and analysis on occurrences of natural EAg within the gill capillaries of sheepshead (*Archosargus probatocephalus*). The ultrastructural morphology of the agglutinated erythrocytes and capillary walls suggested that there was a correlation with infection by myxozoan cysts.

## Materials and Methods

Four specimens of the brackish/marine teleostean fish sheepshead, *Archosargus probatocephalus* (Walbaum, 1792) (Order Perciformes, Family Sparidae), with the Brazilian common name “sargo-de-dente”, were caught in the estuarine region of the Ipioquinha river (9° 30' S; 35° 35' W), in the municipality of Maceió, state of Alagoas, northeastern Brazil, in June 2019, in order to investigate the existence of microparasites (no. 56475-10 MMA/ICMBio). At the time of collection, the following measurements on abiotic parameters were made: salinity 7.0 ± 2.5 ppm; pH 6.2 ± 0.2; temperature 29 ± 1 °C.

The fish were transported alive to the aquaculture laboratory of the Federal University of Alagoas, where they were weighed and measured. The average length was 18 ± 1.5 cm and the average weight was 59 ± 2.5 g. In the laboratory, they were kept alive for about 2-4 hours in an aquarium with aerated brackish water until their dissection. During this period, the behavior of the specimens was observed. Before being dissected, they were anesthetized with MS 222 (100 mg/L) (IACUC, 2020).

Fragments of tissues from different organs (gills, liver, gallbladder, digestive tube, muscles and urinary bladder) were collected for analysis by means of light microscopy (LM). Only the fragments of infected gill filaments containing cysts with myxospores, identified as belonging to the genus *Henneguya* Thélohan, 1892 (Cnidaria, Myxozoa) (study in course), were selected for assessment. Small fragments of infected gill filaments with cysts were fixed in 4% glutaraldehyde in 0.2 M sodium cacodylate buffer (pH 7.2-7.4) for 10-12 h (used for spore fixation, initial objective of the analysis), and were then washed overnight in the same buffer and postfixed with 2% osmium tetroxide in the same buffer for 3 h. All these steps were processed at 4 °C. This was followed by dehydration through an ascending ethanol and propylene oxide series. The material was then embedded in Epon. Semithin sections (1 µm) obtained from the Epon blocks were stained with methylene-Azur II and photographed using an Olympus BX41 light microscope (Olympus, Japan).

The semithin sections assessed through LM (light microscopy) were used to identify the preferentially infected areas of the gill filaments that needed to be sectioned in order to obtain ultrathin sections for observation via TEM (transmission electron microscopy). These ultrathin sections were double-contrasted with uranyl acetate and lead citrate and then observed using a JEOL 100CXII TEM (JEOL Optical, Tokyo, Japan), operated at 60 kV.

## Results

**Figure 1:**
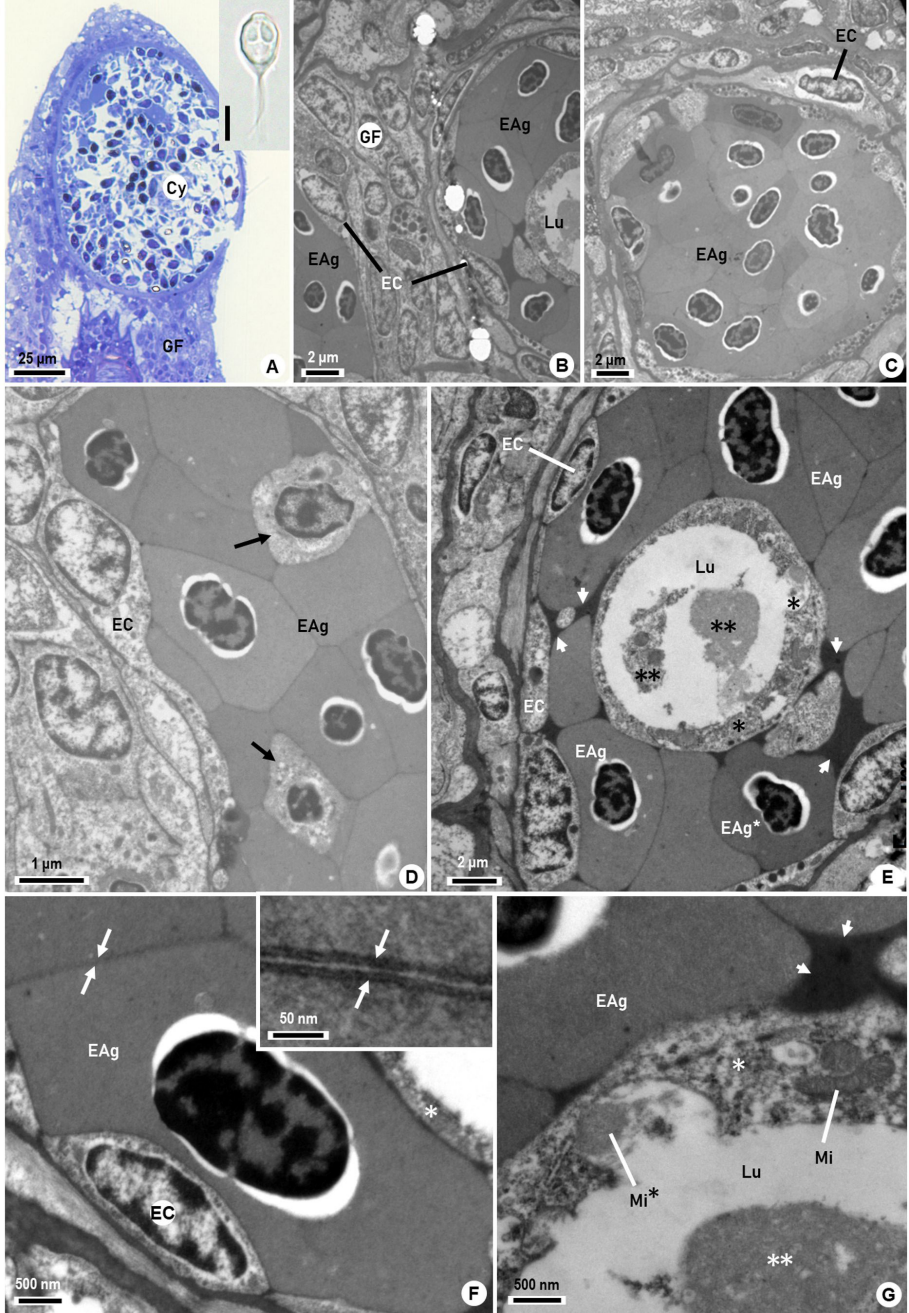
Photomicrograph of a cyst (A) and ultrastructural aspects (B-G) showing phases of the erythrocytic agglutination evolution in capillaries of the branquial filament (GF) of the teleostean *Archosargus probatocephalus*. (A) Semithin section showing a cyst (Cy) with myxospores of *Henneguya* sp., surrounded by host gill tissue (methylene-Azur II). Inset: Morphology of *Henneguya* sp. myxospore showing the polar capsules and the tails observed via LM; (B) Ultrastructural transverse section showing part of two gill capillaries, both displaying agglutinated erythrocytes (EAg) surrounded by endothelial cells (EC) and neighboring cells of the gill filament (GF). One of the capillaries shows that the reduced lumen (Lu) is partially occupied by some amorphous debris; (C) Ultrastructural transverse section of a capillary showing its lumen completely occluded by agglutinated erythrocytes (EAg), in close contact with endothelial cells (EC); (D) Ultrastructural longitudinal section of a capillary region showing the position of the agglutinated erythrocytes (EAg) in close contact with the endothelial cells (EC). Some erythrocytes show the cytoplasm with lysed features (arrows); (E) Ultrastructural transverse section of a capillary showing close adherence among the agglutinated erythrocytes (EAg) surrounded by endothelial cells (EC). The internal space of the capillary lumen (Lu) shows a layer of debris adhering to the erythrocytes (*), along with free amorphous debris of irregular densities (**) located in the reduced capillary lumen. Some erythrocyte cytoplasm with high electron density (EAg*) and dense homogenous structures appeared among the EAg (arrowheads); (F) Ultrastructural transverse section detail of the agglutinated erythrocytes (EAg) showing the close adherence between two erythrocytes (arrows) and endothelial cells of the capillary (EC). In the lumen of the capillary, a small portion of the layer of amorphous mass layer can be observed. Inset: High magnification detailing the adherence between two adjacent erythrocyte cell membranes, showing homogenous intercellular space (arrows); (G) Ultrastructural detail of agglutinated erythrocytes (EAg) in close contact with the dense amorphous masses (arrowheads), the internal layer of the heterogeneous amorphous debris (*) and the internal free debris (**) present in the lumen (Lu). The irregular debris (*) seems to include a mitochondrion (Mi) and an apparent disintegrating mitochondrion (Mi*).

The LM survey conducted in this study revealed some myxozoan cysts infecting the gill filaments of a single fish specimen. These cysts contained numerous myxospores, which were morphologically identified through LM observations (Figure 1A) as belonging to the genus *Henneguya* Thélohan, 1892. This identification was confirmed through TEM observations (Figure 1A, inset). The fishes presented normal movements, without any change in behavior.

Transmission electron microscopic (TEM) observations on the infected tissues further revealed the presence of several agglutinated erythrocytes (EAg) in the gill filament capillaries (Figure 1B-G), which were in close proximity to the cysts. These erythrocytes were ellipsoidal uninucleated cells, with electrodense cytoplasm that did not show any evident organelles (Figure 1C-G). The EAg caused partial or total obstruction of the capillaries, whenever the juxtaposed erythrocytes occupied the totality of the lumen volume (Figure 1B, C). In cases of partial obstruction, the EAg formed a compact block of erythrocytes that occurred adhering to the internal endothelial cells of the capillary walls, thus significantly reducing the lumen volume (Figure 1B-E). One to three layers of EAg displaying irregular thickness were observed in TEM sections that were transverse in relation to the capillary axis (Figure 1D, E). Longitudinal sections of capillaries with EAg showed that the agglutination had the same morphological characteristics reported previously (Figure 1D). The heterochromatin of EAg was well organized in a dense matrix, but with abnormal perinuclear spaces.

The erythrocyte cytoplasm displayed homogeneous electron density and lacked visible cytoplasmic organelles (Figure 1D-F). The EAg had higher electron density (Figure 1D-F), or showed degradation (Figure 1E, F). Some irregular patches of homogeneous denser masses were observed among the EAg (Figure 1E and G). All showed homogeneous close contact between their cytoplasmic membranes, with regular intercellular space of about 85 nm (Figure 1F, inset).

Amorphous masses of irregular texture, in close contact with the agglutinated erythrocytes in the capillary lumen, formed an internal circular ring. These seemed to have resulted from erythrocyte degradation (Figure 1B, E and G). In addition, free irregular masses of varying electron density occurred in the central area of the lumen and seemed similar to the adherent masses located in the reduced space of the capillary lumen (Figure 1E and G). Scarce mitochondria were observed disseminated among the residual heterogeneous material in the capillary lumen (Figure 1E and G). The cells of the endothelium and the cells surrounding the capillaries with EAg showed normal ultrastructural morphology (Figure 1D-F). The endothelial cells in contact with the agglutinated erythrocytes appeared to be linked by tight junctions (Figure 1E, F).

## Discussion

Hemagglutination of erythrocytes is a frequent phenomenon that may occur in species belonging to different groups of vertebrates, under several experimental conditions (Fries, 1986; Gupta & Poddar, 2014; Kurata et al., 2000). It is seen especially in situations of infection by pathogenic agents, such as viruses (Larsen & Mellergaard, 1984; Tamm, 1952; Tavares-Dias et al., 1999; Trust et al., 1981) and microparasites (Yanuhar et al., 2019), or when certain environmental conditions are imposed (Reizenberg et al., 2019).

In the present study, the morphology and structure of the EAg partially or completely filled the gill capillary lumen, as well as being in contact with endothelial cells. This study provides the first report of this occurrence in the lumen of fish in a natural environment.

Alteration of some hematological parameters among different fish species in relation to the environment and infection has been reported in several fish species worldwide (Ahmed et al., 2020; Reizenberg et al., 2019). This may interfere with the metabolism of erythrocytosis, thereby causing erythrocyte agglutination (Ahmed et al., 2020).

Internal biotic factors, such as parasitism, age, sexual maturation cycle or nutritional status, or external abiotic factors, such as temperature, dissolved oxygen, pH, ions, water quality, salinity, environmental pollution or season (Ahmed et al., 2020; Galeotti et al., 2015; Kurata et al., 2000; Yanuhar et al., 2019), interfere with fish metabolism and are major factors responsible for variations in hematological parameters in fish (Ahmed et al., 2020).

It has been reported in the scientific literature that some microparasitic diseases cause hemagglutination in fish (Kurata et al., 2000; Larsen & Mellergaard, 1984; Trust et al., 1981). This has been experimentally correlated with infections by viruses and parasites, including Myxozoa (Kurata et al., 2000; Larsen & Mellergaard, 1984; Trust et al., 1981). In this study, EAg was observed only in the branchial capillaries that were in relative proximity to the Myxozoa cysts. This proximity suggests that the occurrence of hemagglutination may be due to an erythrocyte reaction to the parasitic infection, thus causing partial or total obstruction of the branchial capillary lumen. This finding is supported by a previous study in which the occurrence of hemagglutination in *Cyprinus carpio* experimentally infected with *Myxobolus* sp. was reported (Yanuhar et al., 2019).

The agglutination reaction has also been described in fish infected with other types of parasites. Dash et al. (2014), working with the species *Labeo rohita*, found a high level of serum agglutination in fish parasitized by monogeneans. Other studies involving hemagglutination in fish have been carried out on bacteria, and one of these studies showed that rainbow trout showed hemagglutination in the presence of the bacteria *Aeromonas hydrophila* and *Aeromonas salmonicida* (Trust et al., 1980).

Nonetheless, our observations seem to be the first study reporting the natural occurrence of EAg in fish infected by a myxosporean species. Despite the many studies reporting parasitosis in myxosporean species in other Brazilian fish hosts (Azevedo et al., 2014; Casal et al., 2019; Eiras & Adriano, 2012; Mathews et al., 2016; Rocha et al., 2014), none of these have reported the occurrence of hemagglutination, as a consequence of infection. Moreover, several of the abovementioned studies used LM and molecular procedures and may have missed changes that are only detectable via TEM.

The accumulation of amorphous masses in the capillary lumen space may be due to lysed or degraded erythrocytes that occurs between the EAgs adhering to the capillary walls.

Agglutination causes immobility of the erythrocytes, and the consequent failure of oxidative metabolism of these cells results in their lysis and degradation. Similar disintegration of the erythrocyte membrane giving rise to the appearance of different stages of debris was reported in an experiment using the species *Heteroclarias* sp. (Kori-Siakpere & Ubogu, 2008).

It could be seen that some debris included integral mitochondria, as well as mitochondria in a state of apparent disintegration. Although some authors have reported that the erythrocytes of some species do not have mitochondria (Grosso et al., 2017; Savage, 1983; Youle & Narendra, 2011), others have maintained that the supposed absence of mitochondria in these cells was due to the fact that they were not observed because hemoglobin obscures the presence of these cytoplasmic organelles (Pica et al., 2001; Savage, 1983; Weinreb & Weinreb, 1965). Mitophagy is a known autophagic process of degradation of non-functional cell mitochondria in the maturation phase, as in erythroblasts/erythrocytes, and its existence explains why some fish erythrocytes do not have mitochondria (Grosso et al., 2017; Savage, 1983; Youle & Narendra, 2011), or have a small number of these organelles (Esteban et al., 1989; Pica et al., 2001).

Considering that investigation of EAg was not the main focus of the present study, further analyses should be performed in order to better comprehend how this phenomenon of agglutination occurs in fish capillaries in natural environments and which physicochemical and biological processes may be involved.
